# Chromosome-scale genome assembly and annotation of the two-spotted cricket *Gryllus bimaculatus* (Orthoptera: Gryllidae)

**DOI:** 10.1093/g3journal/jkag036

**Published:** 2026-02-12

**Authors:** Kosuke Kataoka, Ryuto Sanno, Tomasz Gaczorek, Upendra Raj Bhattarai, Yuki Ito, Shintaro Inoue, Kei Yura, Toru Asahi, Guillem Ylla, Taro Mito, Cassandra G Extavour

**Affiliations:** Institute of Engineering, Tokyo University of Agriculture and Technology, 2-24-16 Naka-cho, Koganei-shi, Tokyo 184-8588, Japan; Comprehensive Research Organization, Waseda University, 2-2 Wakamatsu-cho, Shinjuku-ku, Tokyo 162-8480, Japan; Faculty of Biochemistry, Biophysics and Biotechnology, Jagiellonian University, 7 Gronostajowa, Kraków 30-387, Poland; Graduate School of Advanced Science and Engineering, Waseda University, 2-2 Wakamatsu-cho, Shinjuku-ku, Tokyo 162-8480, Japan; Faculty of Biochemistry, Biophysics and Biotechnology, Jagiellonian University, 7 Gronostajowa, Kraków 30-387, Poland; Department of Organismic and Evolutionary Biology, Harvard University, 26 Oxford Street, Cambridge, MA 02138, United States; Harvard T.H. Chan School of Public Health, Harvard University, 677 Huntington Avenue, Boston, MA 02115, United States; Graduate School of Advanced Science and Engineering, Waseda University, 2-2 Wakamatsu-cho, Shinjuku-ku, Tokyo 162-8480, Japan; Bio-Innovation Research Center, Tokushima University, 2272-2 Ishii, Ishii, Ishii-cho, Myozai-gun, Tokushima 779-3233, Japan; Comprehensive Research Organization, Waseda University, 2-2 Wakamatsu-cho, Shinjuku-ku, Tokyo 162-8480, Japan; Graduate School of Advanced Science and Engineering, Waseda University, 2-2 Wakamatsu-cho, Shinjuku-ku, Tokyo 162-8480, Japan; Graduate School of Humanities and Sciences, Ochanomizu University, 2-1-1 Otsuka, Bunkyo-ku, Tokyo 112-8610, Japan; Comprehensive Research Organization, Waseda University, 2-2 Wakamatsu-cho, Shinjuku-ku, Tokyo 162-8480, Japan; Graduate School of Advanced Science and Engineering, Waseda University, 2-2 Wakamatsu-cho, Shinjuku-ku, Tokyo 162-8480, Japan; Faculty of Biochemistry, Biophysics and Biotechnology, Jagiellonian University, 7 Gronostajowa, Kraków 30-387, Poland; Bio-Innovation Research Center, Tokushima University, 2272-2 Ishii, Ishii, Ishii-cho, Myozai-gun, Tokushima 779-3233, Japan; Department of Organismic and Evolutionary Biology, Harvard University, 26 Oxford Street, Cambridge, MA 02138, United States; Department of Molecular and Cellular Biology, Harvard University, 16 Divinity Avenue, Cambridge, MA 02138, United States; Howard Hughes Medical Institute, 4000 Jones Bridge Road, Chevy Chase, MD 20815, United States

**Keywords:** Orthoptera, Gryllidae, two-spotted cricket, white-eyed mutant strain, genome assembly

## Abstract

The two-spotted cricket, *Gryllus bimaculatus*, is a key hemimetabolous model organism for developmental biology, neuroscience, and regeneration. The existing reference genome is, however, highly fragmented into 47,877 scaffolds, hampering chromosome-scale analyses for these fields. Here, we report a high-quality, chromosome-scale genome assembly for the white-eyed mutant strain of this cricket, generated using a combination of Nanopore and PacBio HiFi long reads, integrated with Hi-C data. The final 1.62-Gbp assembly achieves a scaffold N50 of 107.4 Mbp, a significant improvement in contiguity over the previous 6.3-Mbp N50. We anchored 94.45% of the assembly into 15 pseudomolecules, consistent with the known karyotype (*n* = 15). The genome completeness (BUSCO v6.0.0 insecta_odb12) reached 98.1%. We also updated the annotation, identifying 14,964 protein-coding genes. This gene set shows markedly improved completeness (BUSCO v6.0.0 insecta_odb12: 95.7%) compared with the previous annotation (81.2%) and successfully recovers all 9 essential neuropeptide genes previously reported as missing from the draft assembly. This chromosome-scale genomic resource provides an essential foundation for comparative and functional genomics in *G. bimaculatus*.

## Introduction

**Fig. 1.**
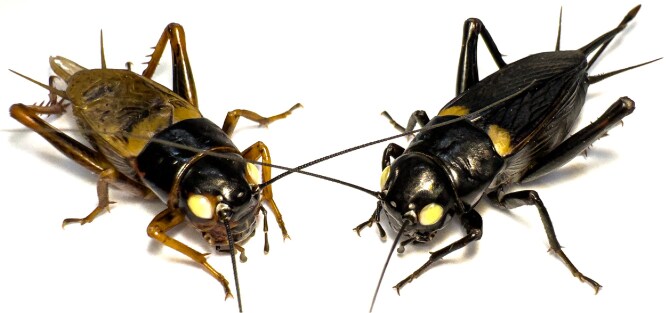
Adult male (left) and female (right) of the white-eyed mutant strain *G. bimaculatus*.

The two-spotted cricket, *Gryllus bimaculatus*, is a model organism for hemimetabolous insects (Horch et al. 2017) (Fig. 1). Unlike holometabolous insects such as fruit fly (*Drosophila melanogaster*) or red flour beetle (*Tribolium castaneum*), hemimetabolous insects undergo direct development, where nymphs hatch and grow through successive molts to become adults without larval or pupal stages. This ancestral developmental mode is crucially important for understanding insect evolution. Due to its ease of rearing, short generation time, and the applicability of efficient RNA interference (RNAi) (Miyawaki et al. 2004) and genome-editing techniques such as CRISPR/Cas9 (Matsuoka et al. 2025), *G. bimaculatus* has been established as a powerful experimental model in a wide range of fields, including evolutionary developmental biology (Donoughe and Extavour 2016), regeneration biology (Nakamura et al. 2008), neuroscience (Matsumoto et al. 2018), and ethology (Abe et al. 2021; Kuriwada 2022). It is also gaining significant global attention as a novel, sustainable food source due to its high protein content and efficient rearing (Kataoka et al. 2020, 2022; Mito et al. 2022).

In the first report of a genomic resource for *G. bimaculatus*, the white-eyed mutant strain, which is standardly used in many functional studies, was sequenced (Ylla et al. 2021). The assembly (GenBank accession: GCA_017312745.1) was a useful resource with a genome size of approximately 1.66 Gb and a scaffold N50 of 6.3 Mb, contributing to analyses of gene family evolution and DNA methylation. However, this previous assembly was fragmented into 47,877 scaffolds and was not assembled to chromosome scale. While chromosome-scale genomes have recently been reported for other related cricket species, such as *Gryllus assimilis* (Ito et al. 2025) and *Acheta domesticus* (Dossey et al. 2023), such a resource has remained unavailable for *G. bimaculatus*, which serves as the primary model for functional, developmental, and neurobiological studies. The lack of a chromosome-scale assembly for this specific species has been a significant limitation for large-scale comparative genomic analyses based on synteny (conservation of gene order), understanding the structural arrangement of transposons and repetitive sequences on chromosomes, and for quantitative trait locus mapping and the accurate identification of genome-editing off-target sites.

In this study, to fill the gap, we constructed a high-quality, chromosome-scale genome assembly using the same white-eyed mutant strain used in the previous study. By combining Nanopore and PacBio HiFi long-read sequencing methods, and Hi-C chromatin conformation capture technology, we anchored 94.45% of the entire genome into 15 pseudomolecules, corresponding to the *G. bimaculatus* karyotype (*n* = 14 autosomes + X) (Yoshimura et al. 2006). This assembly achieves a contig N50 of 4.58 Mb and a scaffold N50 of 107.39 Mb, representing a significant improvement in contiguity. Furthermore, we report an updated gene annotation comprising 14,964 protein-coding genes. This chromosome-scale genome resource will strengthen the foundation for all genomic research using *G. bimaculatus* and accelerate new insights into the biology of hemimetabolous insects.

## Materials and methods

### Animals

A white-eyed mutant strain of *G. bimaculatus* (Mito and Noji 2008) was housed in plastic cases at 30 °C ± 1 °C and 30% to 40% relative humidity under a 10-h light and 14-h dark photoperiod. This strain has been widely used in genetic and developmental studies and was also used in the previous genome assembly, enabling direct comparison with earlier genomic resources (Miyawaki et al. 2004; Ylla et al. 2021). They were nourished with an artificial fish food (4971618–011312, Kyorin, Japan).

### Library preparation and sequencing

A single alive *G. bimaculatus* individual (Fig. 1) was used for genomic DNA extraction. Total genomic DNA was extracted from the head and hind legs of a male *G. bimaculatus* using NucleoBond HMW DNA (Macherey-Nagel, Germany) according to the manufacturer's instructions. This species exhibits an XX/XO sex determination system, in which males are hemizygous for the X chromosome; therefore, a male individual was selected to enable clear identification and chromosome-scale assembly of the X chromosome. The resulting genomic DNA was size selected using a Short Read Eliminator Kit (PacBio, CA, USA). DNA purity and concentrations were measured by spectrometry using NanoPhotometer NP80-TOUCH (Implen, Germany) and fluorometry using Qubit 4 (Thermo Fisher Scientific, MA, USA).

For long-read sequencing, Oxford Nanopore Technologies (ONT) libraries were constructed using the Ligation Sequencing Kit V14 and sequenced on the PromethION 2 Solo platform (Oxford Nanopore Technologies, UK) with a Flow Cell R10.4.1. Base-calling was performed using Dorado v0.3.0 (model: dna_r10.4.1_e8.2_400bps_sup@v4.2.0). The resulting raw reads were adapter trimmed using Porechop_ABI v0.5.0 (Bonenfant et al. 2023), and reads with a mean quality score below Q10 were removed using NanoFilt v2.8.0 (De Coster et al. 2018). Additionally, a SMRTbell library was prepared and sequenced on a PacBio Sequel IIe system.

For chromosome-scale scaffolding, 2 separate Hi-C libraries were prepared. The first library was prepared from the hind legs of a single male *G. bimaculatus* using the Dovetail Omni-C Kit (Dovetail Genomics, CA, USA) following the manufacturer's instructions. The second Hi-C library was generated from the thorax and legs of an adult male using the Proximo Hi-C (Animal) kit (KT2045) (Phase Genomics, Seattle, USA), following the manufacturer's instructions. Both libraries were sequenced on the Illumina NovaSeq 6000 platform, and the sequencing data were combined for downstream scaffolding analysis.

### Genome size estimation

Genome size estimation was performed using ONT reads. A total of 45.0 Gbp of ONT sequencing data was used for k-mer–based analysis. Reads were counted using Jellyfish v2.2.10 (Marçais and Kingsford 2011) with a k-mer size of 21. A k-mer frequency histogram was generated and used as input for GenomeScope v2.0 (Ranallo-Benavidez et al. 2020) to estimate genome size and sequence characteristics. GenomeScope was run with default parameters, and the resulting model was used to infer genome size based on the major k-mer peak.

### Genome de novo assembly

The initial draft genome was assembled by combining the filtered ONT long reads and the PacBio HiFi reads using Flye v2.9.5 (Kolmogorov et al. 2019). To correct errors in the resulting contigs, we retrieved publicly available Illumina short-read data [DDBJ Sequence Read Archive {DRA} accessions DRR272308–DRR272313], which were originally sequenced on an Illumina HiSeq 2000. These reads were downsampled to an approximate 93.9× coverage and used for polishing. The assembly underwent 2 rounds of error correction using POLCA v4.1.0 (Zimin and Salzberg 2020) with default settings.

Potential contamination in the assembly was removed using BlobToolKit v1.1.1 (Challis et al. 2020), which analyzes unexpected coverage, GC content, or similarity to bacterial and other contaminant sequences. Sequence coverage was determined by mapping Illumina reads with bwa v0.7.17-r1188. Similarity analysis was performed using BLASTn v2.13.0 + against NCBI nt database v5 (options: -task megablast culling_limit 10 -evalue 1e-25 -outfmt “6 qseqid staxids bitscore std sscinames sskingdoms stitle”) to assign putative taxonomic origins of contigs as part of contamination screening and assembly quality control. Based on these analyses, no contigs could be confidently identified as apparent contaminants.

The resulting contigs were then corrected for misjoins, ordered, oriented, and anchored into a chromosome-scale assembly using Omni-C data with Juicer v1.9.9 (Durand et al. 2016) and 3D-DNA v180419 (Dudchenko et al. 2017). Candidate assembly was reviewed with Juicebox Assembly Tools v1.9.9 (Durand et al. 2016) for quality control and interactive corrections. The contact map was visualized using Juicebox Assembly Tools v1.9.9. The completeness of the final genome assembly was assessed using BUSCO v6.0.0 (Tegenfeldt et al. 2025) against the insecta_odb12 lineage datasets. In addition, k-mer–based assembly completeness and consensus accuracy were evaluated using Merqury v1.3 (Rhie et al. 2020).

### Mitochondrial genome assembly

The mitochondrial genome was assembled from PacBio HiFi reads using MitoHiFi v2 (Uliano-Silva et al. 2023), following the default workflow. Mitochondrial sequences were excluded from the nuclear genome assembly statistics but are provided as part of the released genome resources for transparency.

### Prediction of repeat regions

In de novo repeat prediction, RepeatModeler v2.0.6 (Flynn et al. 2020) was first used for de novo repeat identification. Because standard libraries are insufficient for effective masking in *Gryllus* genomes (Szrajer et al. 2024), this library was supplemented with the custom repeat library previously generated (Ylla et al. 2021). This custom library was constructed by integrating multiple complementary homology-based and de novo repeat identification approaches, enabling comprehensive annotation of diverse repeat classes (https://github.com/guillemylla/Crickets_Genome_Annotation). The combined repeat library was then used to identify and softmask repetitive elements in the *G. bimaculatus* genome using RepeatMasker v4.2.1 (Smit et al. 2015).

### Structural gene annotation

Structural annotation for protein-coding genes was performed on the softmasked genome using ab initio prediction and RNA-seq-based prediction. Both methods used publicly available RNA-seq data [Sequence Read Archive {SRA} accessions: SRR10619411, SRR10619415, SRR10619417, SRR10619418, SRR10619421, SRR10619423, SRR10619425, SRR10619429, SRR10619431, SRR10619432, SRR10619434, SRR10619437, SRR10619439, SRR10619440, SRR14026720–SRR14026726] as input.

To remove noisy RNA-seq reads potentially arising from erroneous transcription and splicing, de novo transcriptome assembly was first performed using Trinity v2.15.1 (Haas et al. 2013) to generate contigs. The original RNA-seq reads were then mapped back to these contigs using HISAT2 v2.2.1 (Kim et al. 2019) with default parameters, allowing filtration of reads that did not map correctly in the proper orientation as paired-end reads. After removing these noisy reads, the remaining reads were subsequently used for gene predictions.

The ab initio prediction was carried out using BRAKER v3.0.8 (Brůna et al. 2021; Gabriel et al. 2024; Hoff et al. 2016, 2019; Stanke et al. 2008, 2006), incorporating protein data from the OrthoDB 12 arthropods dataset (retrieved from https://bioinf.uni-greifswald.de/bioinf/partitioned_odb12/) and the mapping data of the filtered RNA-seq reads. This BRAKER prediction served as the foundation for our gene set. To complement this base set, StringTie2 v2.2.1 (Kovaka et al. 2019) was used for RNA-seq-based prediction. During this process, StringTie2-derived transcripts were compared against the BRAKER gene models, and only nonredundant transcript classes classified by GffCompare v0.12.6 (Pertea and Pertea 2020) as “u” (intergenic), “i” (intronic), or “y” (contains a reference transcript within its intron) were retained and added to the BRAKER-based gene set. Redundant or overlapping predictions were discarded. This procedure resulted in a final, nonredundant consensus gene set.

### Functional gene annotation

Gene functional annotation was conducted using eggNOG-mapper online (http://eggnog-mapper.embl.de/) (Cantalapiedra et al. 2021) and BLASTp-based methods. For the BLASTp-based annotation, we used databases including *Homo sapiens*, *Mus musculus*, *Caenorhabditis elegans*, *D. melanogaster*, *T. castaneum*, and UniProt Swiss-Prot to identify the best hits for annotation (E-value < 1.0 × 10^−10^).

The completeness of the final predicted gene models was assessed using BUSCO v6.0.0 (Tegenfeldt et al. 2025) against the insecta_odb12 lineage dataset. In addition, annotation quality was further evaluated using OMArk v0.4.1 (Nevers et al. 2025). For these analyses, the longest isoform for each gene was first extracted from the annotation file using AGAT v0.9.1 (Dainat et al. 2022).

### Phylogenomic analysis

We inferred a phylogenetic tree using single-copy orthologs identified by BUSCO (insecta_odb12) from predicted protein-coding genes of *G. bimaculatus* and related insect species. BUSCO analyses were performed using BUSCO v6.0.0, and only single-copy BUSCO proteins shared across all taxa were retained. For each BUSCO ortholog, amino acid sequences were aligned using MAFFT v7.525 (Katoh and Standley 2013) and subsequently trimmed to remove poorly aligned regions with trimAl v1.5.rev1 (Capella-Gutiérrez et al. 2009). The resulting alignments were concatenated into a supermatrix using AMAS v1.0 (Borowiec 2016). A maximum-likelihood phylogenetic tree was then inferred from the concatenated alignment using IQ-TREE v3.0.1 (Wong et al. 2025), with partitioned model selection and ultrafast bootstrap support.

### Validation of neuropeptide gene loci

To assess the completeness of our assembly regarding functionally important gene families, we specifically investigated the neuropeptide gene loci previously reported (Mochizuki et al. 2023). The neuropeptide cDNA sequences listed in the study were retrieved. These sequences were mapped against our final chromosome-scale genome assembly using Exonerate v2.4.0 (Slater and Birney 2005) with the est2genome model to accurately determine exon–intron boundaries. All resulting alignments were manually inspected to validate the gene structures.

## Results and discussion

### Genome sequencing

To construct the genome assembly, we generated 3 types of sequencing data (Table 1). First, we obtained 45.00 Gbp of ONT sequencing data; the average and N50 read lengths were 13.13 Kbp and 24.23 Kbp, respectively. Second, we generated 13.44 Gbp of PacBio HiFi data, with an average read length of 13.61 Kbp and an N50 of 14.17 Kbp. Finally, for chromosome-scale scaffolding, 243.22 Gbp of Hi-C raw data was generated from the Illumina platform.

**Table 1.** Statistics for the DNA-seq data of the *G. bimaculatus* genome.

Platform	Raw data (bp)	Average read length (bp)	N50 Read length (bp)	coverage (X)
ONT	45,004,338,081	13,129.8	24,234	27.73
PacBio HiFi	13,437,781,216	13,611.4	14,170	8.28
Hi-C libraries	243,215,968,945	150	150	149.87

### Genome de novo assembly statistics

Genome size estimation based on Nanopore long-read data using a k-mer–based approach yielded an estimated genome size of approximately 1.64 Gbp (Supplementary Fig. 1). This estimate is highly consistent with the final assembled genome size (1.62 Gbp) and with previous estimates reported for *G. bimaculatus* (Ylla et al. 2021).

The hybrid assembly strategy combining ONT and HiFi reads, followed by 2 rounds of Illumina-based polishing, yielded a 1.63-Gbp draft genome. This polished assembly comprised 3,789 contigs and achieved a contig N50 of 4.58 Mbp (Supplementary Table 1).

**Fig. 2.**
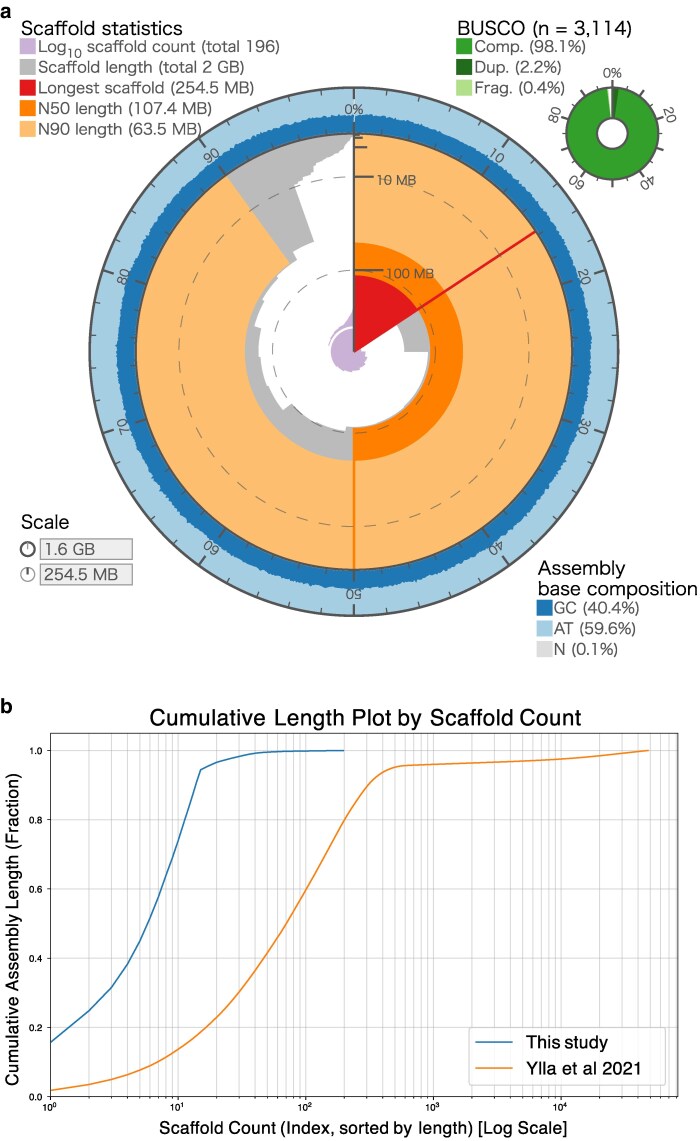
Assembly statistics and contiguity of the *G. bimaculatus* (white-eyed strain) chromosome-scale genome. a) Snail plot visualizing the key statistics of the final assembly. The cumulative assembly length (1.62 Gbp) is plotted in light orange in the middle, with the longest scaffold (254.5 Mbp) shown in red in the center. The the N50 length (107.4 Mbp) is indicated by an orange arc. The inner purple spiral plots the cumulative number of scaffolds (196 total) on a log scale, with white scale lines drawn at successive orders of magnitude from 10 scaffolds onward. The circumferential axis indicates the base composition of the assembly (GC, 40.4%; AT, 59.6%; N, 0.1%). The donut chart (top right) displays the BUSCO completeness (insecta_odb12), showing 98.1% total complete (“Comp.”) genes (light green), which includes 96.0% single-copy and 2.2% duplicated (“Dup.”) genes (dark green). An additional 0.4% were fragmented (“Frag.”) genes (pale green). An interactive version is available at https://kataokaklab.github.io/snailplot-assembly-stats/. b) Cumulative length plot comparing the contiguity of this assembly (blue line on the left) with the previous draft assembly (Ylla et al. 2021) (orange line on the right). The *y* axis shows the percentage of the total genome length covered, and the *x* axis (log scale) shows the number of scaffolds.

Following Hi-C-based scaffolding, the final assembly resulted in a genome of 1.62 Gbp in size, visualized by a snail plot (Fig. 2a). This assembly consists of 196 scaffolds with a scaffold N50 length of 107 Mbp (Table 2). This represents a substantial improvement in contiguity compared to the previous *G. bimaculatus* assembly (Ylla et al. 2021), which had a scaffold N50 of 6.3 Mbp and was fragmented into 47,877 scaffolds (Fig. 2b). To assess genomic completeness, we performed a BUSCO v6.0.0 analysis using the insecta_odb12 dataset. Our assembly achieved a completeness score of 98.1% (C:98.1%[S:96.0%, D:2.2%], F:0.4%, M:1.5%), demonstrating a higher level of completeness compared to the 96.0% (C:96.0%[S:94.3%, D:1.7%], F:1.4%, M:2.6%) of the previous genome (Table 3). A k-mer–based evaluation using Merqury showed that 85.5% of the reliable k-mers derived from the sequencing reads were represented in the genome assembly, with an estimated consensus quality value (QV) of 33.6, corresponding to a base-level accuracy of approximately 99.96%.

**Table 2.** Summary statistics for the chromosome-scale assembly of *G. bimaculatus.*

Features	Statistic
Number of chromosomes	15
Number of scaffolds	196
Scaffold N50 (bp)	107,386,002
GC content (%)	40.36
Max scaffold size (bp)	254,522,992
Total *N* (bp)	1,440,159
Gaps (%)	0.089
Total size (bp)	1,622,898,960

**Table 3.** BUSCO completeness assessment of the *G. bimaculatus* genome assembly.

BUSCO v6.0.0 (insecta)	This study	Ylla et al. 2021
Complete BUSCOs	98.1%	96.0%
Single-copy complete BUSCOs	96.0%	94.3%
Duplicated complete BUSCOs	2.2%	1.7%
Fragmented BUSCOs	0.4%	1.4%
Missing BUSCOs	1.5%	2.6%

A total of 15 pseudochromosomes were identified based on Hi-C-guided scaffolding using 3D-DNA and accounted for 94.45% of the total genome length (Table 4). This number (*n* = 15) is in agreement with the established karyotype for *G. bimaculatus* (*n* = 14 autosomes + X), which was previously determined by cytogenetic analysis (Yoshimura et al. 2006), and is also consistent with that of the recently reported species *G. assimilis* (Ito et al. 2025), which is closely related to *G. bimaculatus*. The X chromosome was identified as the longest, accounting for 15.68% of the genome, which is in accordance with the karyotype of this species (Yoshimura et al. 2006). This was further validated by the observation that the X chromosome displayed half of the read coverage compared to the autosomal chromosomes, calculated using a genomic short-read library (DRR272308) from a single hemizygous male (Supplementary Fig. 2).

**Table 4.** Statistics of the assembled pseudochromosomes in *G. bimaculatus*.

Chromosome	Length (bp)	Proportion in genome
chrX	254,523,492	15.68%
chr1	148,286,872	9.14%
chr2	109,203,633	6.73%
chr3	109,044,855	6.72%
chr4	108,018,790	6.66%
chr5	107,387,002	6.62%
chr6	100,738,338	6.21%
chr7	98,895,268	6.09%
chr8	83,461,245	5.14%
chr9	81,413,916	5.02%
chr10	78,899,412	4.86%
chr11	69,666,237	4.29%
chr12	66,393,650	4.09%
chr13	63,483,624	3.91%
chr14	53,360,289	3.29%
Total	1,532,776,623	94.45%

Repetitive sequence annotation revealed that 823.1 Mbp of the genome, corresponding to 50.72% of the assembled sequence, is composed of repetitive elements (Table 5). This includes a substantial contribution from both Class I retroelements (13.95%) and Class II DNA transposons (10.53%), as well as a notable fraction of unclassified repeats (18.15%). The proportion of annotated repetitive sequences is markedly higher than that reported in the previous *G. bimaculatus* assembly, in which only 33.69% of the genome was annotated as repetitive (Ylla et al. 2021), likely reflecting improved assembly contiguity in the present study.

**Table 5.** Classification of repetitive sequences of the *G. bimaculatus* genome assembly.

Type	Length (bp)	Proportion in genome (%)
Retroelements	226,335,103	13.95
SINEs	20,383,703	1.26
Penelope	1,718,510	0.11
LINEs	156,279,698	9.63
LTR elements	47,953,192	2.95
DNA transposons	170,814,501	10.53
Unclassified	294,555,500	18.15
Total interspersed repeats	691,705,104	42.62
Rolling-circles	20,709,593	1.28
Other repeats	113,971,824	7.02
Total	823,137,644	50.72

### Gene annotation

The final consensus gene set, derived from merging BRAKER ab initio predictions and StringTie2 RNA-seq-based predictions, comprised 14,964 protein-coding genes (Table 6). This total is less than the 17,871 genes reported previously (Ylla et al. 2021), likely because the improved scaffold contiguity of our assembly allows for the correct assembly of genes previously split across multiple scaffolds. Of the 14,964 predicted genes, functional annotation was assigned using eggNOG-mapper and BLASTp (E-value < 1.0 × 10^−10^) against several model organisms’ annotation datasets. eggNOG-mapper annotated 71.96% of the genes. The BLASTp searches (E-value < 1.0 × 10^−10^) against databases including *H. sapiens*, *M. musculus*, *C. elegans*, *D. melanogaster*, *T. castaneum*, and UniProt Swiss-Prot yielded hit rates ranging from 44.84% to 75.76% (Table 7).

**Table 6.** Summary statistics for gene prediction in *G. bimaculatus*.

Features	
Number of protein-coding genes	14,964
Mean exons per mRNA	7.0
Mean gene length (bp)	29,659
Mean exon length (bp)	2,32
Mean intron length (bp)	4,696

**Table 7.** Structural statistics of the *G. bimaculatus* gene set.

Annotation sources	
*H. sapiens* (GRCh38)	44.84%
*M. musculus* (GRCm39)	71.89%
*C. elegans* (WBcel235)	55.18%
*D. melanogaster* (BDGP6.32)	67.16%
*T. castaneum* (icTriCast1.1)	75.76%
UniProt/Swiss-Prot (release: 2020_06)	66.74%
eggNOG-mapper	71.96%

The completeness of this annotation set was validated using BUSCO v6.0.0. The analysis identified 95.7% of the expected complete insecta BUSCOs (C:95.7%[S:93.6%, D:2.0%], F:1.5%, M:2.9%) (Table 8). OMArk analysis indicated a completeness of 92.67% and a consistency score of 71.54% (Supplementary Table 2). Together, these independent metrics support the overall quality of the predicted gene models.

**Table 8.** Functional annotation summary for the *G. bimaculatus* gene set.

BUSCO v6.0.0 (insecta)	This study	Ylla et al. 2021
Complete BUSCOs	95.7%	81.2%
Single-copy complete BUSCOs	93.6%	79.9%
Duplicated complete BUSCOs	2.0%	1.3%
Fragmented BUSCOs	1.5%	8.9%
Missing BUSCOs	2.9%	10.0%

We next inferred a maximum-likelihood phylogeny using single-copy BUSCO orthologs shared across *G. bimaculatus* and closely related orthopteran species (Supplementary Fig. 3). The resulting topology places *G. bimaculatus* within Gryllidae and in the expected relationship to other *Gryllus* and cricket lineages, providing an additional consistency check and a comparative framework for future evolutionary analyses using this updated genome resource.

### Recovery of missing neuropeptide genes

A recent comprehensive study (Mochizuki et al. 2023) highlighted significant gaps in the previous draft genome assembly (Ylla et al. 2021). They reported that several crucial neuropeptides genes [e.g. ACP {adipokinetic hormone/corazonin-related peptide}, allatotropin, and kinin] were missing from the draft genome and could only be identified within de novo transcriptome assemblies, suggesting these loci were absent from the previous reference.

We asked if our new chromosome-scale assembly was more complete in this regard, by mapping the cDNA sequences of these previously missing neuropeptides. We successfully located all 9 of these genes encoding neuropeptides [i.e. ACP, allatostatin CC {Ast CC}, allatotropin, CCHamide-1, CCHamide-2, CRF/DH {corticotropin-releasing factor-like diuretic hormone}, kinin {leucokinin}, neuropeptide F1a {NPF1a}, neuropeptide F1b {NPF1b}], which are now correctly anchored onto our pseudomolecules. For example, the ACP gene, previously missing, was successfully mapped to chromosome X, where it spans 11,668 bp and is composed of 3 exons, revealing its complete exon–intron structure (Supplementary Fig. 4). This demonstrates that our assembly not only improves contiguity to the chromosome scale but also recovers functionally critical genes that were absent in the previous reference, providing a more complete and reliable resource for functional genomics in *G. bimaculatus*.

### Conclusions

We have generated a high-quality, chromosome-scale genome assembly and updated gene annotation for the key hemimetabolous model organism *G. bimaculatus*. This assembly represents a substantial upgrade to the previous draft sequence (Ylla et al. 2021), increasing the scaffold N50 from 6.3 to 107.4 Mbp and anchoring 94.45% of the sequence into 15 pseudomolecules, consistent with the known karyotype (Yoshimura et al. 2006).

Crucially, our assembly resolves significant gaps present in the previous version, evidenced by the recovery of 9 essential neuropeptide genes previously reported as missing (Mochizuki et al. 2023). This improved completeness is further supported by superior BUSCO scores for both the genome (98.1% vs 96.0%) and the gene set (95.7% vs 81.2%). This highly contiguous and complete genome sequence provides an essential new foundation for the *G. bimaculatus* research community, facilitating advanced genetic and genomic analyses, such as synteny comparisons, QTL mapping, and the precise design of genome-editing experiments.

## Supplementary Material

jkag036_Supplementary_Data

## Data Availability

The scripts used for the analyses in this study are available in GitHub (https://github.com/Kataoka-K-Lab/Gryllus_bimaculatus_genome_gbim_v2.2). All bioinformatics tools used in this study followed their respective manuals and protocols. The software versions, codes, and parameters are provided in the Materials and Methods section. Unless otherwise specified, default parameters were used. The assembled genome and annotation datasets are available in Figshare (https://doi.org/10.6084/m9.figshare.30472754.v1) (Kataoka 2025). The genomic WGS sequencing data were deposited in the NCBI SRA database under the BioProject PRJNA1347939. All data and assembly statistics reported in this study correspond to the version before contamination screening and removal performed by NCBI. Supplemental material available at *G3* online.
