# Supplementary material for: Chromosome-scale genome assembly and annotation of the two-spotted cricket Gryllus bimaculatus (Orthoptera: Gryllidae)
Source: G3 (Bethesda). 2026 Feb 12;16(4):jkag036. doi: 10.1093/g3journal/jkag036 (PMC13042277; doi:10.1093/g3journal/jkag036)
Supplement: jkag036_Supplementary_Data [file jkag036_supplementary_data.zip › Supplemental_Figures_G3-2025-406355.docx]

**Supplementary Figures**


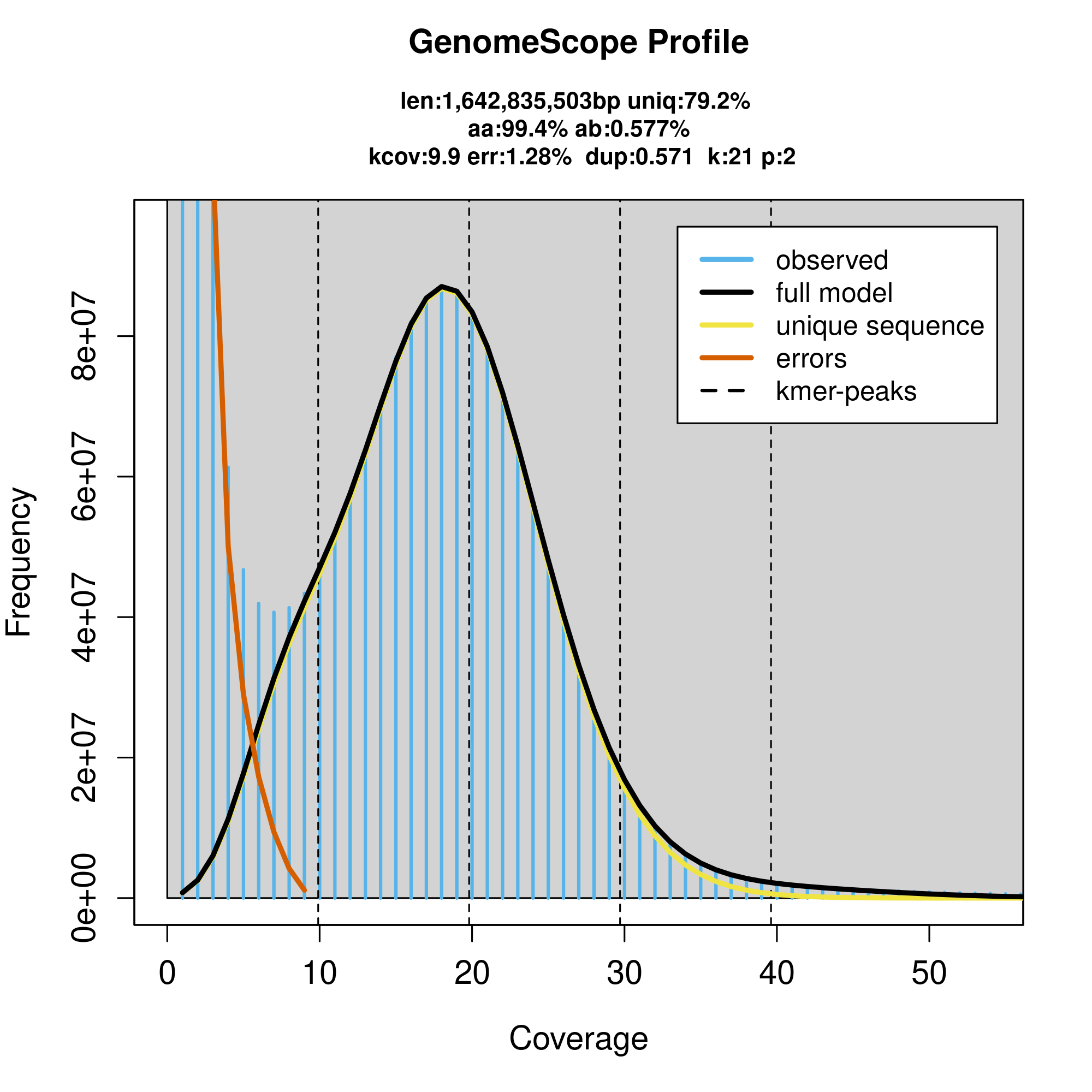


**Figure S1. Genome size estimation of *Gryllus bimaculatus* based on ONT long-read data.**

A k-mer frequency histogram (k = 21) generated from ONT long-reads was analyzed using GenomeScope. The model estimates a genome size of approximately 1.64 Gbp.

**
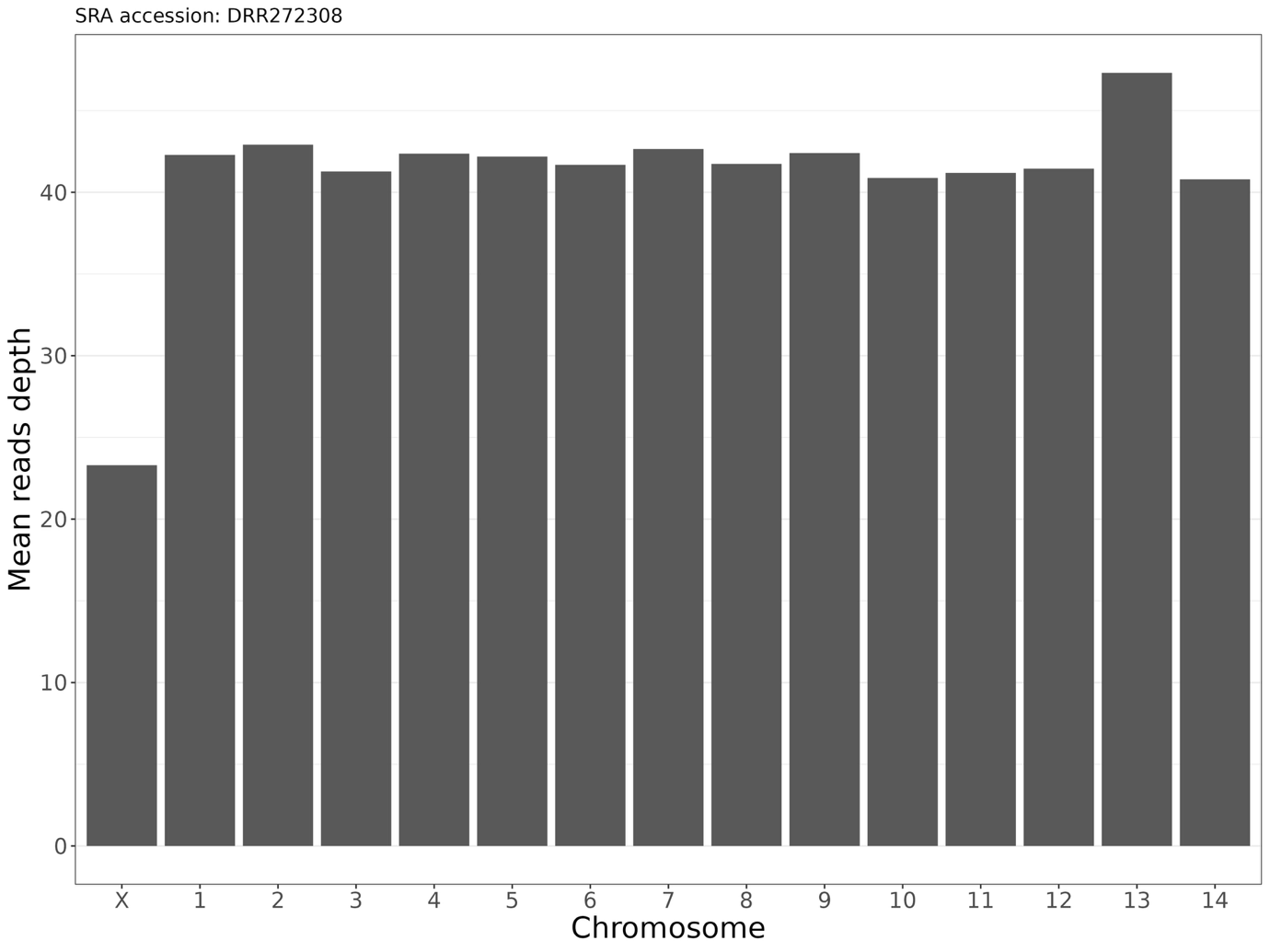
**

**Figure S2. Genomic read coverage confirms X chromosome hemizygosity.**

Mean read coverage depth across all assembled chromosomes derived from the mapping of genomic short reads of a single male *Gryllus bimaculatus* individual (SRA: DRR272308). The X chromosome displays approximately half the average autosomal coverage depth, consistent with the expected pattern of male hemizygosity.

**Figure S3. BUSCO-based phylogeny of *Gryllus bimaculatus* and related insects**

Single-copy BUSCO orthologs shared across all included taxa were extracted (insecta_odb12), aligned at the amino-acid level, trimmed, concatenated into a supermatrix, and used to infer a maximum-likelihood phylogeny with partitioned model selection. Node support values indicate bootstrap support.


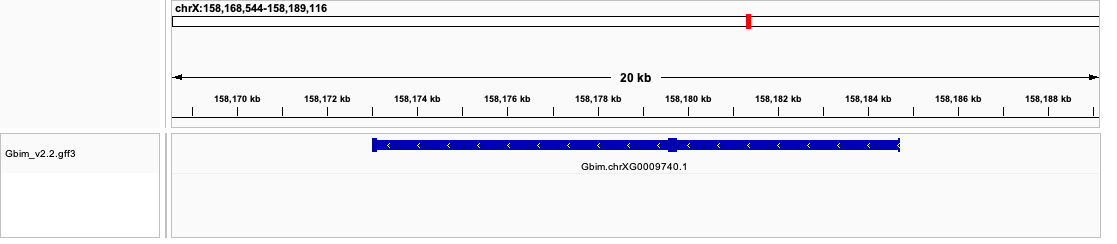


**Figure S4. Recovery of the Adipokinetic hormone/corazonin-related peptide (ACP) gene, previously missing from the draft genome.**

The image displays an Integrative Genomics Viewer (IGV) screenshot of the *G. bimaculatus* chromosome-scale assembly. The complete gene model for ACP (Gbim.chrXG0009740.1), one of the nine neuropeptide genes reported missing from the first assembly report (Mochizuki et al., 2023), is shown. The gene is now successfully anchored and annotated on Chromosome X (chrX), spanning a region of approximately 20 kb. The blue track (Gbim_v2.2.gff3) shows the full exon-intron structure (exons as thick blocks, introns as thin lines).
